# Study of Visual Evoked Potentials in Schoolchildren: A Promising Aid to Pediatric Ophthalmology

**DOI:** 10.7759/cureus.67813

**Published:** 2024-08-26

**Authors:** Ruchi Kothari, Sujay Srivastava, Azhar Sheikh, Ashay Gomashe, Alind Murkhe, Naveenkumar Nallathambi, Suryadev Vrindavanam, Prashanth A

**Affiliations:** 1 Physiology, Mahatma Gandhi Institute of Medical Sciences, Wardha, IND; 2 Ophthalmology, Mahatma Gandhi Institute of Medical Sciences, Wardha, IND; 3 Internal Medicine, Central Government Health Scheme, Ministry of Health and Family Welfare, Nagpur, IND; 4 Internal Medicine, Madras Medical College, Chennai, IND

**Keywords:** schoolchildren, refractive error, p100 amplitude, p100 latency, visual evoked potential

## Abstract

Background

Visual evoked potential (VEP) is a noninvasive investigation conducted to identify abnormalities in the visual system. It is especially suitable for young children who are unable to express visual symptoms or participate in conventional vision tests. This study was undertaken to examine the VEP among schoolchildren to assess the functionality of their optic pathway.

Methodology

This short-term observational study was performed in the Clinical Neurophysiology Unit of the Physiology Department of a rural medical college. The study population consisted of 60 schoolchildren aged 7-12. Both eyes were examined for transient pattern reversal VEP recordings using a Recorders & Medicare Systems Electromyography-Evoked Potential recorder (RMS EMG-EP MARK-II Pvt. Ltd., Chandigarh, India).

Results

VEPs were analyzed for latency and amplitude of the main components, namely P100, N70, and N155. The results showed markedly extended P100 latency in 33.33%, i.e., eight out of 24 eyes of standard (std.) III children. Similar latency prolongation was obtained in 36.36% (eight out of 22) eyes of std. IV, 30% (six of 20 eyes) of std. V, 13.63% (three of 22 eyes) of std. VI, and 50% (eight of 16 eyes) in std. VII and VIII children. A markedly reduced P100 amplitude was observed in two of 20 eyes (10%) of std. V, two of 16 eyes (12.5%) in std. VII and VIII children, amounting to a P100 amplitude abnormality in 5% eyes in toto. The interocular differences in all VEP parameters among the subjects were statistically insignificant.

Conclusion

In schoolchildren in whom normal latencies and amplitudes were obtained, the presence of reproducible VEPs indicated the normal functional status of their visual pathway. On the other hand, in those children where altered VEP findings were found, it hinted toward complementary information that they may have underlying ocular disorders that were yet to be diagnosed. Hence, this study provides insight into the assessment of visual system function, which is primarily difficult in young children.

## Introduction

Visual evoked potential (VEP) is an objective, noninvasive technique to investigate the nervous pathway extending from the eyes to the brain's visuosensory areas. It is similar to an electroencephalogram (EEG) in that it records the brain's electrical activity. However, it differs in focusing specifically on the parts of the brain that involve vision, i.e., the occipital cortex [1]. The test records the responses generated in one's brain while he or she is visualizing a TV monitor on which a black-and-white checkerboard pattern is generated. This technique demonstrates whether or not correct information about what a child's eyes can visualize is received by the brain [2]. VEP is an investigation that can provide useful information about the functioning of the visual system. It is a simple, rapid, clinically tested, and objective noninvasive tool that is especially useful for pediatric populations who cannot describe their visual complaints or are less compliant with typical vision tests. VEP studies are highly beneficial for many pediatric patients, such as those with sensory visual pathway disorders or those who might be at risk of damage to their visual pathway [2,3].

Electrophysiological evaluation incorporating VEPs has been employed for timely diagnosis, planning of future therapies, and preventive strategies in children with cortical visual impairment, a predominant cause of visual deficit worldwide [3,4]. It has been documented in the past that there is a significant correlation between abnormal VEPs and delayed latency and diminished amplitude in children with the presence of global developmental delay and suffering from spastic cerebral palsy [5]. Although VEPs demonstrate brisk visual maturation until age seven, further enhancement occurs more steadily during the early years of growth. Preadolescent school life for a child is marked by notable alterations in brain anatomy and physiology regarding visual function [6]. Therefore, comparing a child's VEPs at various ages during childhood is reasonable. An altered VEP in schoolchildren may yield objective evidence of an unpredicted or suspected but unproven anomaly.

Out of the two commonly used modalities for recording VEP for children, the checkerboard pattern reversal technique gives the greatest amplitude of the prominent wave at approximately seven to eight years since it has been documented that up to six years, the brain attains 90% of its adult dimension [7]. Reproducible VEPs in schoolchildren have been recorded in 15 boys and 17 girls within the age range of 6-12 years using transient and steady-state VEPs by Tomoda et al., and they confirmed the use of pattern reversal as a stimulus for evaluation in children being superior to flash light-emitting diodes (LEDs) [8]. Marr et al. retrospectively reviewed 112 children of less than 10 years with high myopia and 114 consecutive children under 10 years with high hyperopia to investigate the influence of refractive error found during childhood on VEPs. They reported a strong association of refractive errors in early childhood with ocular disorders, which lead to poor reading and writing skills and eventually hamper children's academic performance [9,10].

Although some studies [11-13] have been conducted in the past to record pattern reversal VEPs (PRVEPs) in adolescents, they reported mixed findings regarding the characteristics of VEP peaks across ages. Also, there is a considerable variation in the latency and the size of the most prominent P100 wave of PRVEP among previous studies [8-10], apart from the lack of data regarding the other two waves, namely N70 and N75. Particularly in schoolchildren, evaluating the normative data of all waveform parameters and understanding the developmental trajectory of VEPs is crucial for their application in diagnosing and managing visual and neurological disorders. Hence, this study was undertaken for a cohort of schoolchildren to explore the characteristics and clinical implications of VEPs along with the neurophysiological basis behind visual impairment, if any, found in them to prove VEP as a potential aid to pediatric ophthalmologists to pick up and detect early defects in the visual pathway.

## Materials and methods

Study design and setting

This was a short-term observational study in the Clinical Neurophysiology Unit of the Physiology Department of a rural medical college in central India. The Institutional Ethics Committee approved the study before commencement (Ref. No. MGIMS/IEC/PHY/57/2017). The parents of all study participants provided written informed consent.

Study population

A total of 60 children in the age range of 7-12 years constituted the study population. The study included VEP responses from both the eyes of the participants. The subjects were recruited using a convenient sampling method. Children with 6/6 best corrected visual acuity (BCVA), no significant history of known visual disorders, and those who voluntarily gave informed consent were included in the study. Those with significant optic nerve disorders, such as miosis, cataracts, glaucoma, <6/6 BCVA, amblyopia, use of mydriatic or cycloplegic drugs in the last 12 hours, any disorder that could affect the performance of VEP, and a history of head injury or recent neurosurgery, were excluded from the study.

Data sources and measurement of variables

Data were collected in a structured proforma from all participants. Anthropometric variables, such as height, weight, and occipitofrontal circumference, were estimated using standard procedures. A thorough ophthalmological examination was conducted for all participants, including an evaluation of the BCVA. VEP recordings were performed on both eyes using the Recorders & Medicare Systems Electromyography-Evoked Potential recorder (RMS EMG-EP MARK-II Pvt. Ltd., Chandigarh, India). The standard protocol established by the International Federation of Clinical Neurophysiology (IFCN) Committee Recommendations [14] and the International Society for Clinical Electrophysiology of Vision (ISCEV) Guidelines [15] was followed for performing the investigation. The montages followed the 10-20 International System of EEG Electrode Placements [16].

The stimulus employed for VEP in this study utilized the transient pattern reversal method, where a black-and-white checkerboard pattern was generated, covering the full visual field, and presented on a VEP monitor using an electronic pattern generator. Each square in the checkerboard subtended a visual angle of 2.2° while the entire pattern spanned 18° at the eyes of the participant. The checkerboard display consisted of an equal number (eight) of white-and-black squares produced by the Visual Basic software (Microsoft Corporation, Redmond, Washington), with a red square fixation point centrally positioned at the intersection of four squares. The pattern reversal occurred at a frequency of 1 Hz, with a recording sensitivity of 2 μV. A sweep duration was set at 300 ms, and less than 5 KΩ electrode impedance was maintained throughout the test. A total of 200 stimuli were presented, and evoked responses were amplified and averaged for each eye. Each eye was tested with two trials. The setting of pattern luminance was 59 cd/m², and the contrast was 80%. Electrical signals were filtered across a band spread of 2-100 Hz.

A PRVEP waveform consisted of the first negative peak arriving at 70 ms (N70), followed by a prominent positive peak appearing at 100 ms (P100), succeeded by the second negative peak (N155) at a latency of 155 ms.

Statistical analysis

The mean and standard deviation of all the VEP parameters were evaluated. The data analysis was performed using IBM SPSS Statistics for Windows, Version 25 (Released 2017; IBM Corp., Armonk, New York). The level of significance was kept at a p-value of <0.05.

## Results

VEPs were recorded in 60 schoolchildren aged 7-12. They were grouped according to the class in which they were studying. The students were recruited from standards (std.) III to VIII (Table 1).

**Table 1:** Subject distribution

S. No.	Class	No. of Subjects (Out of 60)	Percentage
1	III	12	20%
2	IV	11	18.3%
3	V	10	16.6%
4	VI	11	18.3%
5	VII	8	13.3%
6	VIII	8	13.3%

VEPs were analyzed for latency and amplitude of the main components, namely P100, N70, and N155, among all the study participants. Table 2 shows the VEP parameters of the left and right eyes of the total 60 study subjects. The mean values were well within the normal ranges of the reference normative data of the Clinical Neurophysiology Unit.

**Table 2:** VEP parameters of the left and right eyes of the study subjects (n=60) VEP: visual evoked potential; SD: standard deviation; N70: negative wave appearing at a latency of 70 ms; P100: positive wave appearing at a latency of 100 ms; N155: negative wave appearing at a latency of 155 ms; ms: milliseconds; µV: microvolts

S. No.	VEP Parameter	Values of the Left Eye (Mean ± SD)	Values of the Right Eye (Mean ± SD)
1	N70 (ms)	71.15 ± 4.17	72.41 ± 4.6
2	P100 (ms)	104.71 ± 3.5	105.49 ± 4.27
3	N155 (ms)	158.88 ± 9.91	158.98 ± 10.58
4	N70-P100 (µV)	12.89 ± 5.23	11.59 ± 4.80

Table 3 tabulates the VEP latencies of the left and right eyes in individual class groups. The table depicts that maximum P100 latency was found in std. VIII for the left eye and std. VII for the right eye.

**Table 3:** VEP latencies of the left and right eyes of each study group VEP: visual evoked potential; SD: standard deviation; N70: negative wave appearing at a latency of 70 ms; P100: positive wave appearing at a latency of 100 ms; N155: negative wave appearing at a latency of 155 ms; ms: milliseconds

Class	N70 Latency (ms) (Mean ± SD)		P100 Latency (ms) (Mean ± SD)		N155 Latency (ms) (Mean ± SD)	
	Left	Right	Left	Right	Left	Right
III	70.33 ± 4.55	73.60 ±3.64	103.68 ± 3.35	105.84 ± 3.08	155.95 ± 12.38	160.24 ± 15.19
IV	72.92 ± 2.92	73.92 ± 3.75	104.82 ± 2.78	106.63 ± 2.49	159.14 ± 7.75	163.53 ± 9.77
V	69.68 ± 5.93	71.08 ± 7.57	104.33 ± 4.12	106.59 ± 5.71	157.02 ± 12.30	159.82 ± 10.56
VI	70.54 ± 4.17	71.83 ± 2.50	103.53 ± 3.46	102.83 ± 3.54	162.91 ± 8.88	156.93 ± 8.48
VII	73.01 ± 3.22	71.45 ± 5.27	106.03 ± 3.36	107.22 ± 3.34	161.33 ± 9.75	159.75 ± 6.20
VIII	70.73 ± 5.86	71.99 ± 4.11	106.89 ± 3.60	103.98 ± 6.16	157.23 ± 6.56	151.85 ± 7.60

**Figure 1:**
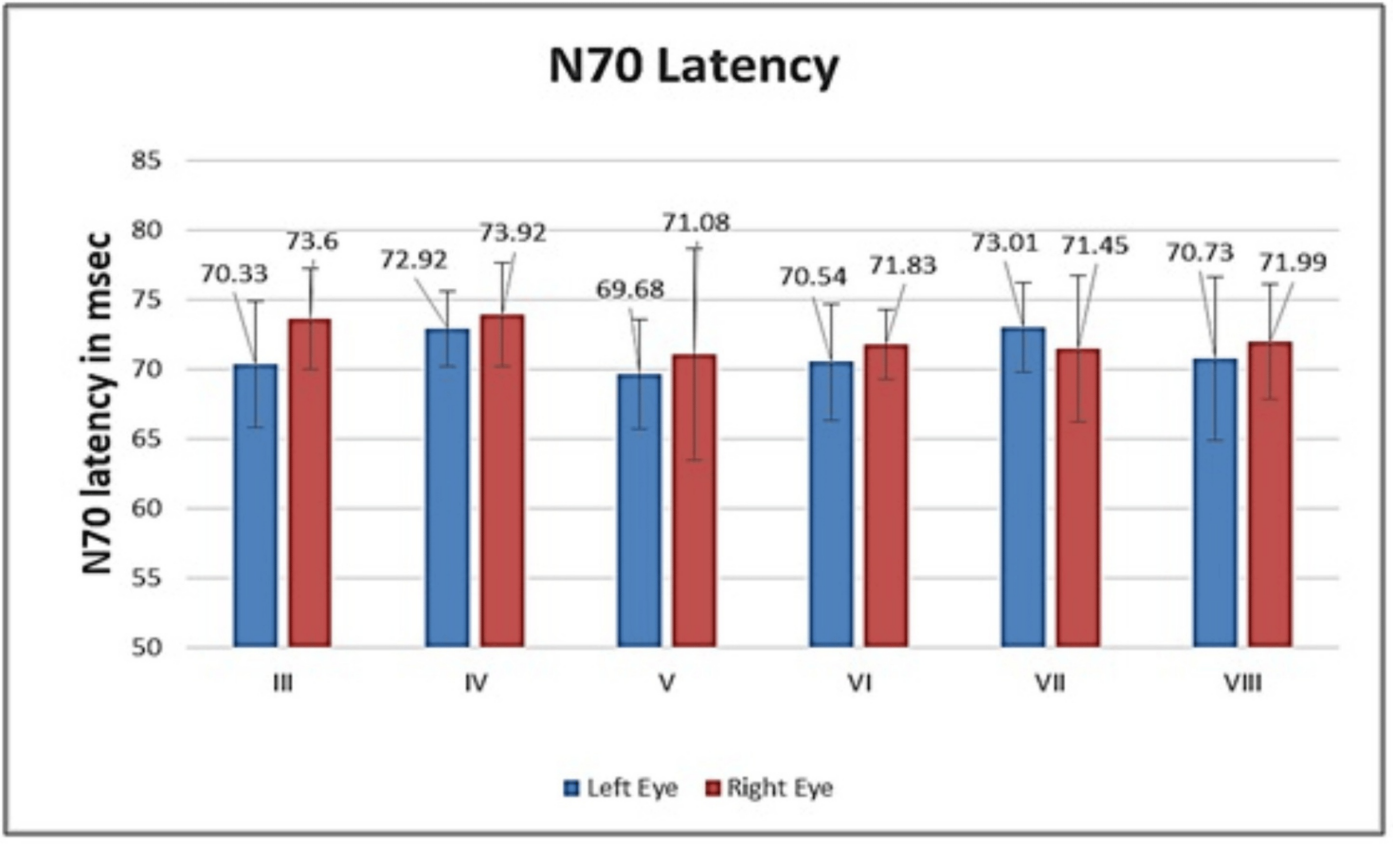
Graphical representation of N70 latency of both eyes for all study groups

**Figure 2:**
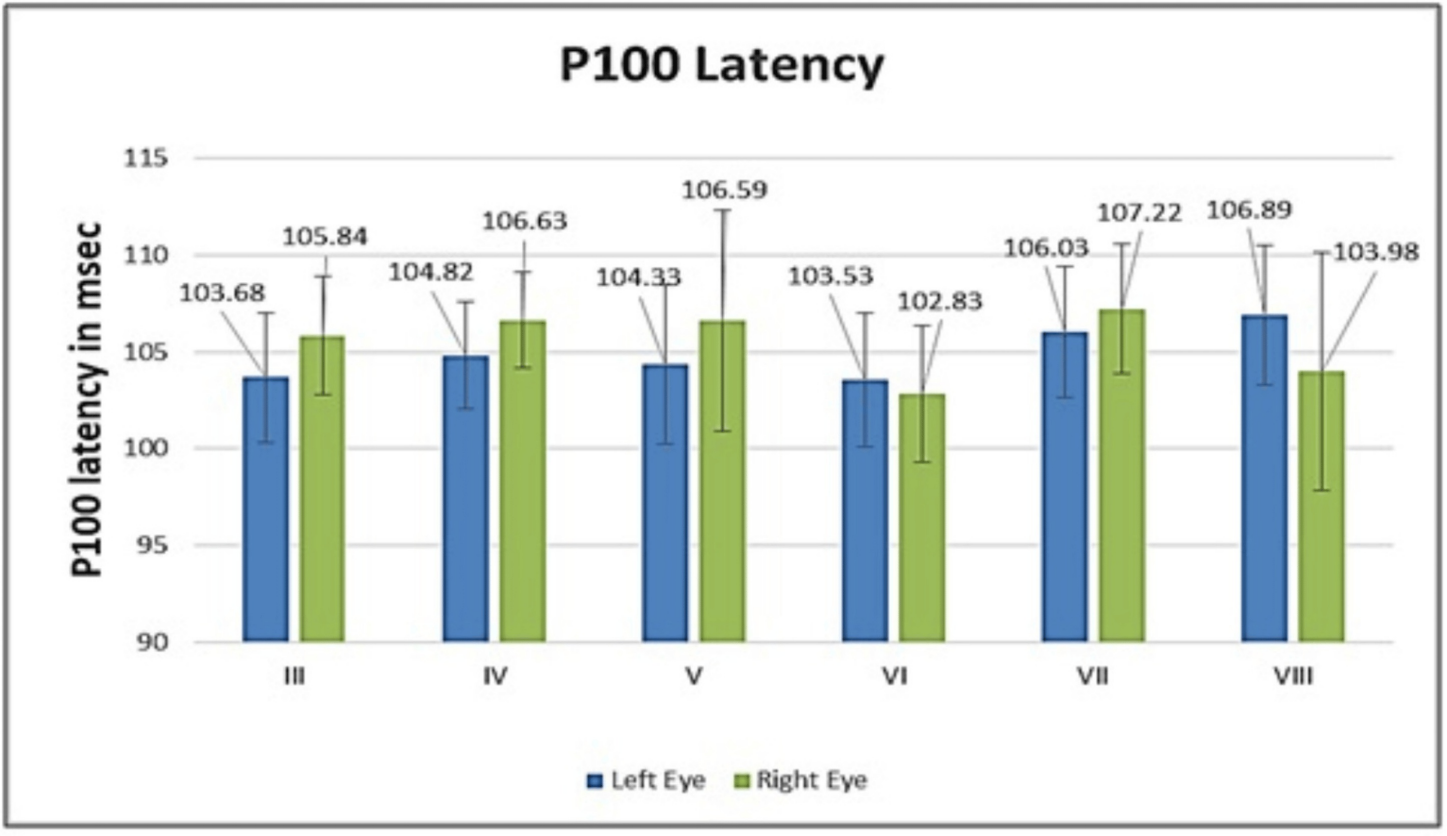
Graph depicting the P100 latency of both eyes for all study groups

**Figure 3:**
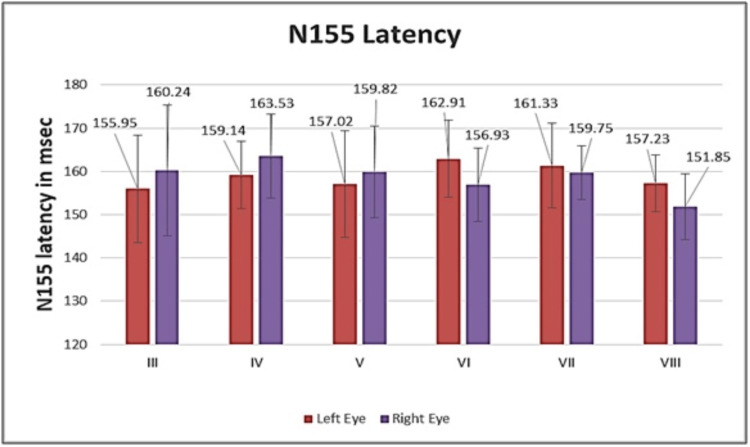
Graphical representation of N155 latency of both eyes for all study groups

The graphical representation for both eyes of all study groups is shown in Figure 1 for N70 latency, Figure 2 for P100 latency, and Figure 3 for N155 latency. It is evident from the figures that there was no significant inter-eye difference between the groups with regard to N70, P100, and N155 latencies.

**Figure 4:**
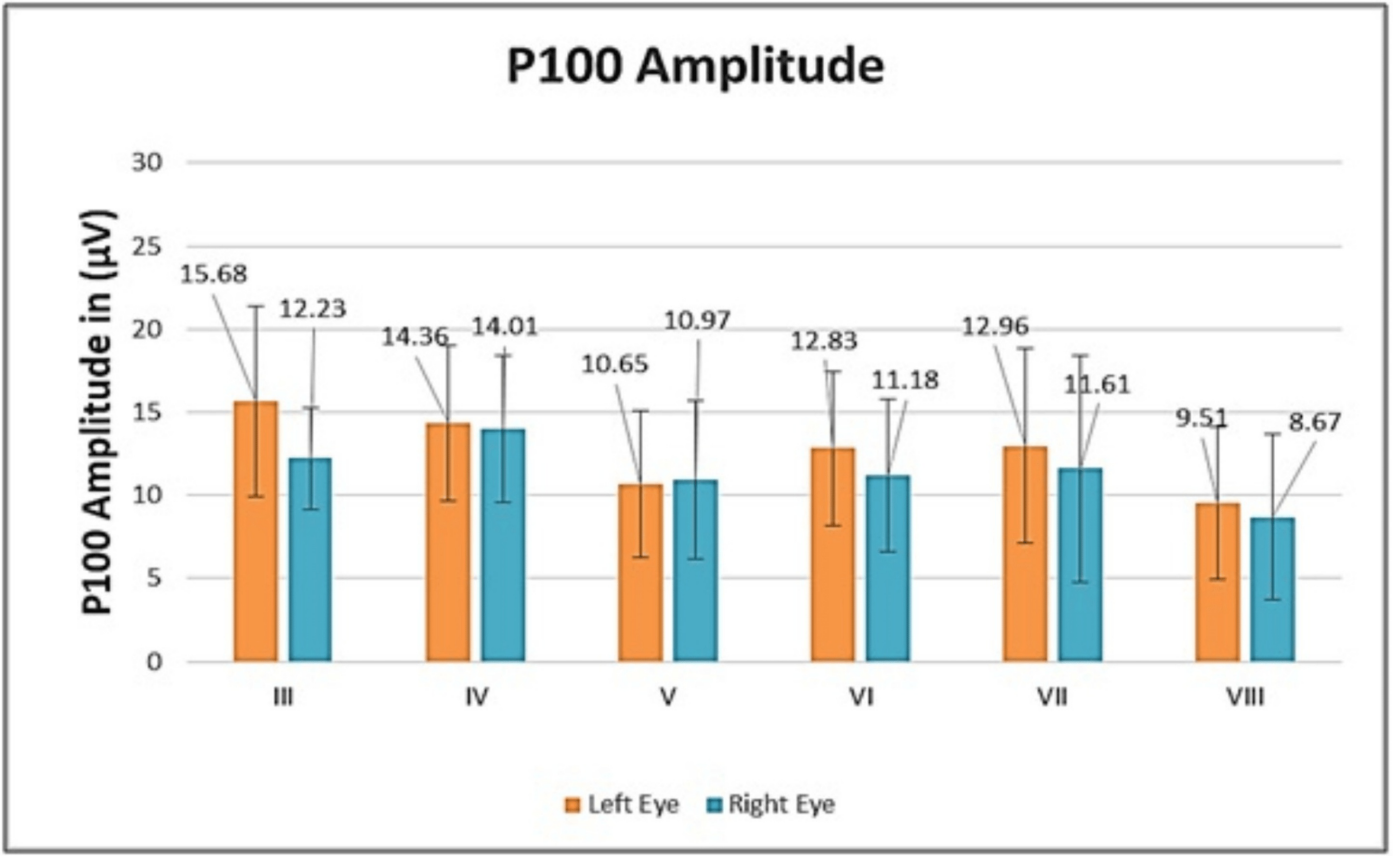
Graphical representation of P100 amplitude of both eyes for all study groups

Table 4 depicts the P100 amplitude of the left and right eyes in individual class groups, and its graphical representation is illustrated in Figure 4. The values in the table and figure clearly show that the highest amplitude was found in the youngest children of the study population, i.e., of std. III, as compared to the remaining groups.

**Table 4:** VEP P100 amplitude of the left and right eyes of each study group VEP: visual evoked potential; SD: standard deviation; N70: negative wave appearing at a latency of 70 ms; P100: positive wave appearing at a latency of 100 ms; µV: microvolts

S. No.	Class	N70-P100 (µV) (Mean ± SD)	
		Left	Right
1	III	15.68 ± 5.74	12.23 ± 3.07
2	IV	14.36 ± 4.66	14.01 ± 4.44
3	V	10.65 ± 4.43	10.97 ± 4.76
4	VI	12.83 ± 4.61	11.18 ± 4.58
5	VII	12.96 ±5.86	11.61 ± 6.84
6	VIII	9.51 ± 4.58	8.67 ± 4.98

Upon statistical analysis by unpaired Student's t-test, there was a statistically significant (p<0.05) difference between the groups of Class III with V (p=0.034) and Class III with VIII (p=0.017) regarding the P100 amplitude.

Table 5 displays the inter-eye differences in the VEP parameters of the study subjects' left and right eyes. As the interocular differences of each VEP parameter among the children were statistically insignificant and found to be in the normal range, it rules out the presence of any major monocular abnormality in the 120 eyes investigated for VEPs.

**Table 5:** Interocular difference of the VEP parameters VEP: visual evoked potential; IOD: interocular difference; N70: negative wave appearing at a latency of 70 ms; P100: positive wave appearing at a latency of 100 ms; N155: negative wave appearing at a latency of 155 ms; ms: milliseconds; µV: microvolts

S. No.	VEP Parameter	IOD (Left Eye to Right Eye)
1	N70 (ms)	1.27
2	P100 (ms)	0.78
3	N155 (ms)	0.10
4	N70-P100 (µV)	1.30

The results of the study showed a markedly extended P100 latency in 33.33%, i.e., eight out of 24 eyes of std. III children. Similar latency prolongation was obtained in 36.36% (eight out of 22) eyes of std. IV, 30% (six of 20 eyes) of std. V, 13.63% (three of 22 eyes) of std. VI, and 50% (eight of 16 eyes) in std. VII and VIII children. A markedly reduced P100 amplitude was observed in two of 20 eyes (10%) of std. V and two of 16 eyes (12.5%) in std. VII and VIII children, amounting to a P100 amplitude abnormality in 5% of eyes in toto.

## Discussion

Timely identification and prompt treatment of visual disorders in children are essential to prevent permanent vision loss [12]. Most of the time, it is difficult to ascertain how much the schoolchildren can see. In other words, vision assessment in young children is quite difficult through a standard eye chart. They are also very helpful if the ophthalmologist cannot find an obvious reason why a child sees poorly. It offers the pediatric ophthalmologist a measurable indicator of visual deficit. It may serve to predict a decline in subjective visual acuity much earlier than it becomes evident during a clinical examination [2,3].

The present study was conducted on schoolchildren to investigate their VEP responses using pattern reversal. No statistically significant difference was obtained among various school standards regarding N70, P100, and N155 latencies. Our observations of VEP latencies align with those of Mahajan and McArthur, who analyzed VEPs among 90 adolescents utilizing three types of pattern reversal stimuli [11]. They suggested that significant changes in visual processing pathways occur in the adolescent period. The amplitudes of N75 and P100 waves were reduced, but N135 was enlarged, and their peaks' latencies demonstrated no evident change with age. Tobimatsu and Celesia have also shown by their qualitative analysis of the VEP waveform that P100 was consistently present at all ages [7].

Our study found a statistically significant difference between Class III with V (p=0.034) and Class III with VIII (p=0.017) about P100 amplitude. Recently, Thompson et al. studied 2750 pattern VEPs investigated in children ages 16 and below and found the appearance of giant VEPs having an amplitude of P100 wave ranging from 65 to as huge as 163 μV in 27 cases, 81% of which had a risk of raised intracranial tension (ICT). They highlighted the significance of VEPs at normal latency but with sustained giant amplitude as a clue to raised ICT [12]. As per the results of the present study, most of the eyes had VEP latencies and amplitudes within the normal reference range as set up in our Clinical Neurophysiology Unit. However, 41 out of 120 eyes, i.e., 34.16%, had markedly prolonged P100 latency, which suggests a critical delay in the impulse conduction along the visual pathway up to the occipital cortex. The probable cause of delay might be an uncorrected refractive error, which has remained obscured. Another reason that could be implicated after taking a history of delayed latencies from the students was the irregular use and non-compliance in wearing glasses, even after detecting a refractive error. As it is known from a previous study, since VEP is heavily representative of the foveal function, it is more sensitive to even minor refractive changes [17].

Additionally, there was a marked reduction of P100 amplitude in six of 120 eyes, suggesting axonal loss. This could be attributed to some ocular disorder affecting the health of the axons in the visual pathway, which needs to be identified through a thorough ophthalmological examination. In childhood, PRVEPs in girls are usually documented [13,18] to be a little faster than boys (2 or 3 ms at 4-11 years) owing to their smaller head size and occipitofrontal circumference, which was recognized as a general attribute in VEPs of girls in our cohort as well in comparison to boys' PRVEPs. VEP recording also differentiates visual deficit from visual inattention in pediatric subjects [3,4]. A child's performance in VEP tests relies on skills they have mastered, so it is crucial to predict any deficits early and provide appropriate, timely intervention targeting those specific deficits, helping to minimize the long-term effects of the disability.

Limitations

This study's limitations should be carefully considered when interpreting its findings, especially in the context of its application to pediatric ophthalmology. As the study involved children of a younger age range and VEP testing requires adequate compliance, it was challenging to ensure a smooth conduction of the investigation. Like any other cross-sectional study, it provides only a snapshot of the pediatric VEP responses at a specific time. This limits the understanding of how VEPs might evolve, which is crucial in pediatric populations where development is rapid. This also makes it difficult to understand the progression of visual impairments or the long-term effectiveness of interventions. Schoolchildren vary in the developmental stage, and factors such as socioeconomic backgrounds and health conditions might influence VEPs. Without longitudinal follow-up, it is challenging to account for such differences.

## Conclusions

Reproducible VEPs with pattern reversal stimulus configuration were obtained in children aged seven to 12. The presence of normal VEP latencies and amplitudes in most schoolchildren indicated the normal functional status of their visual pathway. On the other hand, in those children where altered VEP findings were found, it hinted toward complementary information that they may have underlying ocular disorders, which were yet to be diagnosed. Hence, this study provides insight into assessing visual system function, which is primarily difficult in young children.
